# Convolutional auto-encoder for image denoising of ultra-low-dose CT

**DOI:** 10.1016/j.heliyon.2017.e00393

**Published:** 2017-08-30

**Authors:** Mizuho Nishio, Chihiro Nagashima, Saori Hirabayashi, Akinori Ohnishi, Kaori Sasaki, Tomoyuki Sagawa, Masayuki Hamada, Tatsuo Yamashita

**Affiliations:** a Clinical PET Center, Institute of Biomedical Research and Innovation, 2-2, Minatojimaminamimachi, Chuo-ku, Kobe, Hyogo 650-0047, Japan; b Division of Molecular Imaging, Institute of Biomedical Research and Innovation, 2-2, Minatojimaminamimachi, Chuo-ku, Kobe, Hyogo 650-0047, Japan; c Department of Radiology, Kobe University Graduate School of Medicine, 7-5-2 Kusunoki-cho, Chuo-ku, Kobe, Hyogo 650-0017, Japan

**Keywords:** Computer science, Medical imaging

## Abstract

**Objectives:**

The purpose of this study was to validate a patch-based image denoising method for ultra-low-dose CT images. Neural network with convolutional auto-encoder and pairs of standard-dose CT and ultra-low-dose CT image patches were used for image denoising. The performance of the proposed method was measured by using a chest phantom.

**Materials and methods:**

Standard-dose and ultra-low-dose CT images of the chest phantom were acquired. The tube currents for standard-dose and ultra-low-dose CT were 300 and 10 mA, respectively. Ultra-low-dose CT images were denoised with our proposed method using neural network, large-scale nonlocal mean, and block-matching and 3D filtering. Five radiologists and three technologists assessed the denoised ultra-low-dose CT images visually and recorded their subjective impressions of streak artifacts, noise other than streak artifacts, visualization of pulmonary vessels, and overall image quality.

**Results:**

For the streak artifacts, noise other than streak artifacts, and visualization of pulmonary vessels, the results of our proposed method were statistically better than those of block-matching and 3D filtering (p-values < 0.05). On the other hand, the difference in the overall image quality between our proposed method and block-matching and 3D filtering was not statistically significant (p-value = 0.07272). The p-values obtained between our proposed method and large-scale nonlocal mean were all less than 0.05.

**Conclusion:**

Neural network with convolutional auto-encoder could be trained using pairs of standard-dose and ultra-low-dose CT image patches. According to the visual assessment by radiologists and technologists, the performance of our proposed method was superior to that of large-scale nonlocal mean and block-matching and 3D filtering.

## 1 Introduction

Advances in CT technology have improved image quality and increased the total number of CT examinations. However, this has raised concerns about radiation exposure and the potential cancer risk induced by it [1]. To limit the radiation dose, low-dose CT (LDCT) was performed in clinical situations. For example, National Lung Screening Trial confirmed a 20% reduction in lung cancer mortality among subjects allocated to a LDCT screening group [2].

Although LDCT has proven useful for screening of lung cancer, the cumulative radiation dose associated with LDCT is a major problem. McCunney et al. showed that the cumulative radiation dose from LDCT screening could exceed that received by nuclear workers or atomic bomb survivors if LDCT lung cancer screening was conducted over a 20–30-year period [3]. They also showed that such a radiation dose could independently increase the risk of lung cancer beyond that associated with cigarette smoking.

To overcome this issue, ultra-low-dose CT (ULDCT) has been studied intensively [4, 5, 6]. Because images obtained by LDCT or ULDCT are severely affected by noise (e.g., streak artifact) and differentiation between normal/abnormal findings on the noisy CT images is difficult, image-processing techniques have been utilized to improve image quality of LDCT or ULDCT [4, 5, 6, 7, 8, 9, 10]. While many methods have been proposed in previous studies, image-processing techniques of LDCT or ULDCT can be roughly divided into two categories: raw-data-based techniques [4, 5, 6] and post-processing techniques [7, 8, 9, 10]. In the present study, we focused on post-processing techniques. Although the major strength of post-processing techniques is that it can be applied directly to CT images, it is often difficult to differentiate noise and artifacts from the actual signal on the noisy images. For example, Chen et al. showed that although one type of large-scale nonlocal mean (LNLM) [7, 11] was useful for denoising abdominal LDCT images, the LNLM method was not effective in suppressing the non-stationary streak artifacts in thoracic CT images [8].

One previous study showed that it was possible to achieve state-of-the-art image-denoising performance with plain multilayer perceptron that maps noisy image patches onto noise-free ones [12]. The performance of this perceptron could rival that of block-matching and 3D filtering (BM3D) [13], a well-engineered image-denoising algorithm. In line with this trend, the present study utilized neural network for image denoising of ULDCT images as a post-processing technique. One approach to use neural network as image denoising is denoising auto-encoder (DAE), a special type of neural network. DAE takes a pair of original input and noisy input, maps the noisy input to the latent representation, and uses the latent representation to reconstruct the output [14]. DAE trains its parameter such that the loss between the original input and its reconstruction is reduced. While noise was artificially added to the original input in the previous study of DAE, pairs of standard-dose CT (SDCT) and ULDCT image patches were used to train DAE in the present study. In addition, we utilized convolutional auto-encoder (CAE) [15] to improve image denoising. If CAE is successfully trained with pairs of SDCT and ULDCT image patches, CAE would output the noise-free image patch of ULDCT.

The objectives of the present study were: i) to validate a patch-based, neural-network-trained image-denoising method for ULDCT images; ii) to train the neural network with CAE and pairs of SDCT and ULDCT image patches; and iii) to investigate the performance of our proposed method using a chest phantom. In addition, our proposed method was compared with the method proposed by Chen et al. [16].

## 2 Materials and methods

The present study was experimental, and phantom study was performed. No patient information was used in the present study.

### 2.1 CT scan of chest phantom

Helical CT scans of chest phantom (PBU-X-21; Kyoto kagaku, Japan) were acquired from lung apices through lung bases using a 16-detector row scanner (lightspeed; GE Healthcare, USA). During a CT examination session, one set of SDCT images and two sets of ULDCT images were obtained. One set of UDLCT images was used for training, while the other was used for testing. The following CT parameters were used: tube current, 300 mA (SDCT) or 10 mA (ULDCT); tube potential, 120 kV; and gantry rotation time, 0.5 s. Raw CT data were reconstructed into 1.25-mm-thick images using high-frequency kernel. The CT scanner was calibrated regularly.

### 2.2 Denoising auto-encoder

**Fig. 1:**
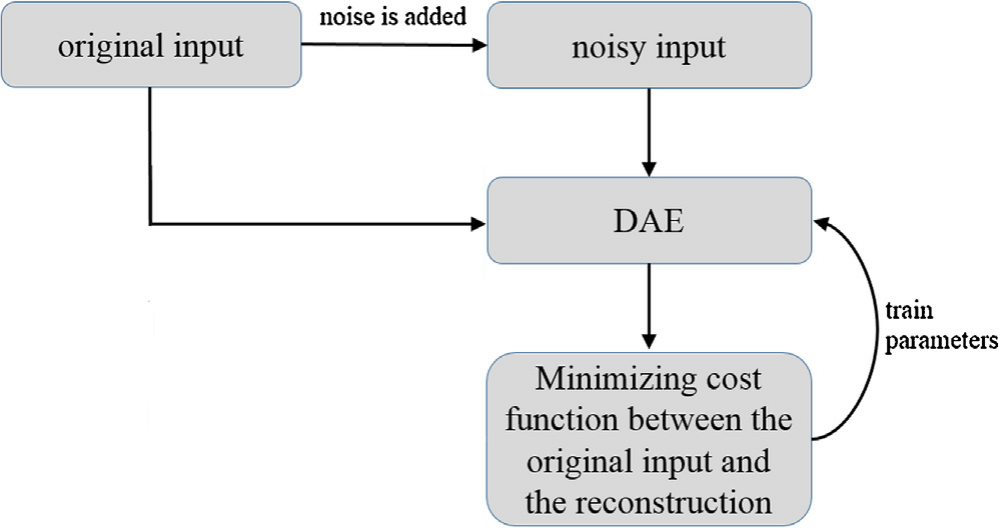
Schematic illustration of training the conventional DAE. Abbreviation: DAE, denoising auto-encoder.

DAE [14] is a special type of 3-layered neural network comprising an input layer, one-hidden layer, and an output layer. First, the data set *D_n_* = {***x_1_***, …, ***x_n_***} is prepared, where ***x_i_*** is a d-dimensional vector. Then, noise is added to ***x_i_*** (e.g., Gaussian noise), and pairs of original input and noisy input, *T_n_* = {(***x_1_***, ***y_1_***), …, (***x_n_***, ***y_n_***}}, are constructed, where ***y_i_*** is obtained by adding noise to ***x_i_***. DAE takes ***y_i_*** and maps it to the latent representation ***h_i_*** using a function ***h_i_*** = *σ* (***W y_i_*** + ***b***) with parameters {***W***, ***b***}. Here, σ is an activation function, and the sigmoid function was used in our main experiment. Then, the latent representation ***h_i_*** is used to reconstruct the clean output ***x_i_***’ by reverse mapping ***x_i_***’ = *σ* (***W***’ ***h_i_*** + ***b***’) with parameters {***W***’, ***b***’}, where the first parameter is constrained as ***W***’ = ***W***^T^. This constraint means that the same weights are used for encoding the input and decoding the latent representation. The parameters are optimized, minimizing an appropriate cost function between ***x_i_*** and ***x_i_***’ (*i* = 1, …, *n*) over the training set *T_n_*. If the training is successfully performed, DAE reconstructs the clean output from the noisy input. A schematic illustration of the training DAE is shown in Fig. 1.

### 2.3 Convolutional auto-encoder

Although the DAE ignores the 2D-image structure, CAE preserves the spatial locality of the 2D image [15]. While the architecture of CAE is similar to that of DAE, the major difference between DAE and CAE is that i) the weights are shared and ii) CAE takes a 2D image as its input. First, pairs of original and noisy images, *T_n_* = {(***x_1_***, ***y_1_***), …, (***x_n_***, ***y_n_***)}, are given as training set to CAE. For a noisy image ***y*** (here, index ***i*** is omitted), the latent representation of the *k*-th feature map is given by ***h^k^*** = σ (***y*** ∗ ***W^k^*** + ***b^k^***), where ∗ denotes the 2D convolution and ***W^k^*** is the weight of the *k*-th feature map. The reconstruction ***x***’ is obtained using ***x***’ = $\sigma \left(\underset{k\in H}{\sum }h^{k}*W{\text{'}}^{k}+c\right)$, where ***c*** is a bias parameter, *H* identifies the group of latent feature maps, and ***W***’ identifies the weights. The parameters of CAE are trained, minimizing a cost function. In the present study, the cost function of CAE is the mean squared error (MSE) between ***x_i_*** and ***x_i_***’ (*i* = 1, …, *n*) over the training set *T_n_*: $MSE=\frac{1}{n}\overset{n}{\underset{i=1}{\sum }}{\left(x_{i}-x{\text{'}}_{i}\right)}^{2}$. After the training, CAE can reconstruct the clean 2D image from the noisy 2D image. In the present study, CAE was implemented using Python (http://www.python.org/) and chainer (http://www.chainer.org/).

### 2.4 Preprocessing of CT images and preparation of the training set

**Fig. 2:**
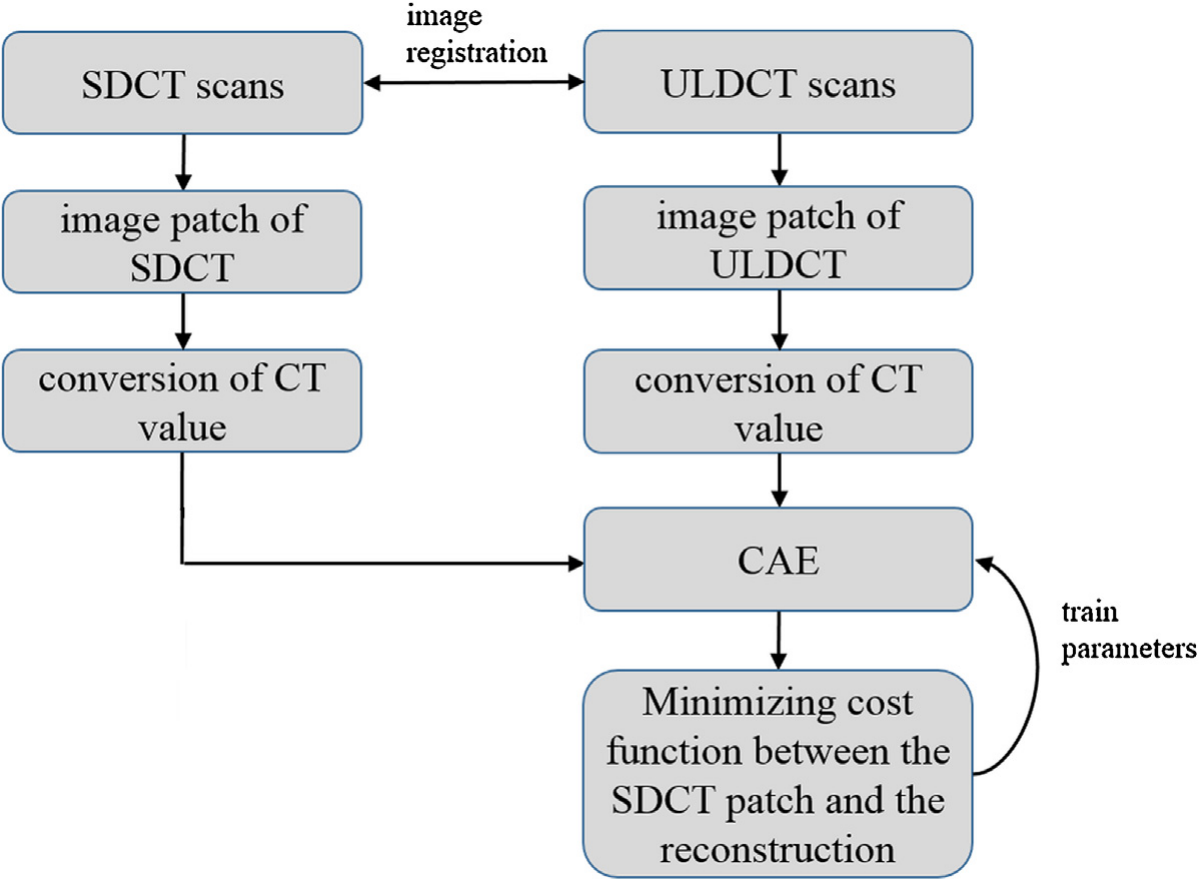
Schematic illustration of the preprocessing of CT images and training CAE. Abbreviation: CAE, convolutional auto-encoder; SDCT, standard-dose CT; ULDCT, ultra-low-dose CT.

Before training CAE, patches of the SDCT and ULDCT images were prepared. First, the SDCT and training ULDCT images were three-dimensionally co-registered using Advanced Normalization Tools [17] because misregistration between the SDCT and ULDCT images was caused by the difference in CT scan path. Then, pairs of image patches with sizes = 28 × 28 pixels were randomly extracted from lungs of the SDCT and training ULDCT images, and 100,000 pairs were obtained for training. Here, pairs of patches of SDCT and ULDCT images were represented as *T_n_* = {(***x_1_***, ***y_1_***), …, (***x_n_***, ***y_n_***)}, where ***x_i_*** and ***y_i_*** are patches obtained from SDCT and ULDCT images, respectively. Next, for ease of training, the CT value of each pixel in the image patches was converted to results of the following function *f(x)*:

[I] $$f\left(x\right)=\{\begin{array}{c} 0\text{ i}\text{f}\;x\;<-\;1500\\ 1\text{ if}\;x\;>\;1500\\ (x\;+\;1500)\;/\;3000\text{ otherwise} \end{array}$$

where *x* is CT value. After converting the CT value, *T_n_* was used for training of CAE. As stated, the number of patches was 100,000 in the training set. A schematic illustration of the preprocessing of CT images and the training of CAE is shown in Fig. 2. To evaluate the effectiveness of the training, other pairs of image patches were obtained from the SDCT and training ULDCT images (these pairs are referred to as validation set). The number of patches was 10,000 in validation set.

### 2.5 Architecture of CAE and its training

**Fig. 3:**
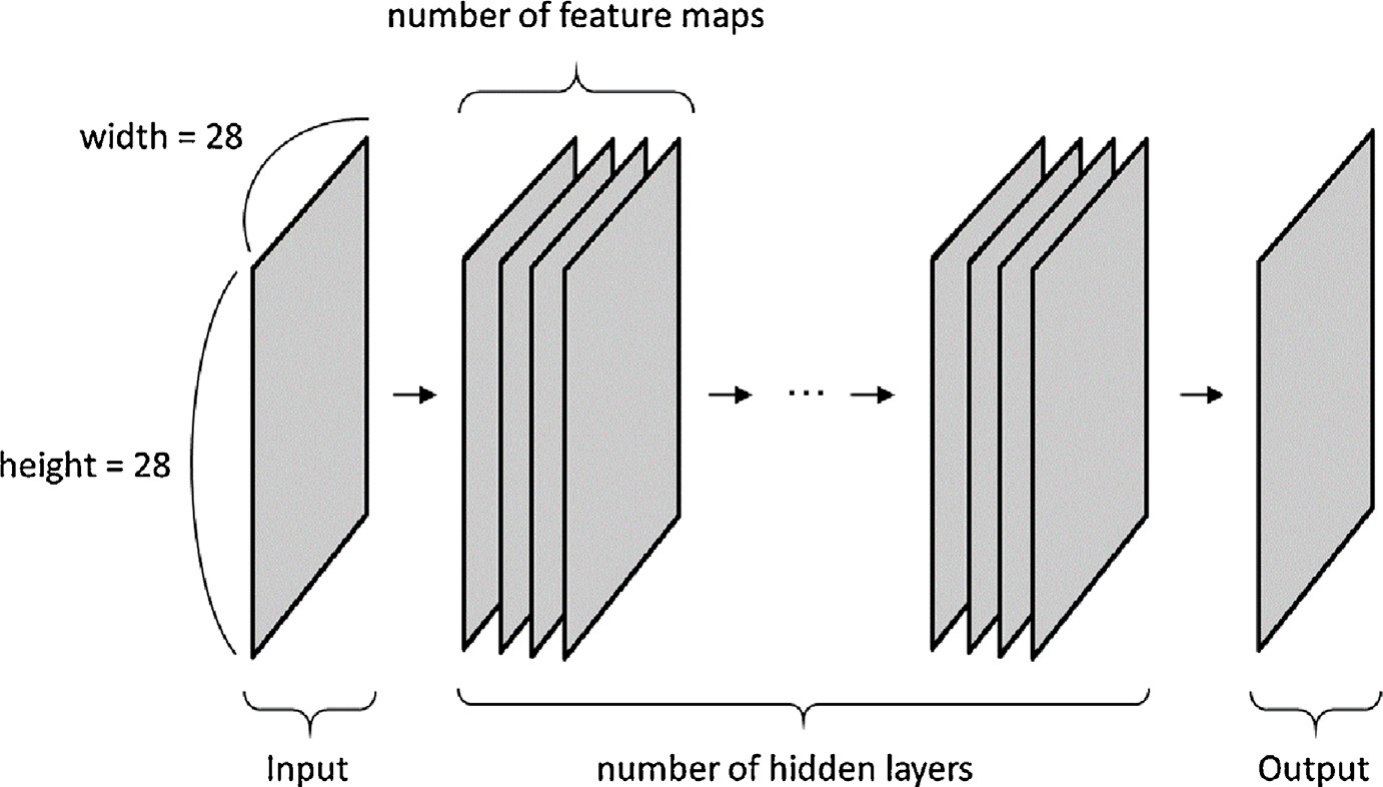
Network architecture of CAE with multiple hidden layers. Note: CAE with multiple hidden layers corresponds to conventional convolutional network without a pooling layer. In the current study, the number of hidden layers ranged from 1 to 11. The number of feature maps were 20, 30, and 40. To maintain the sizes of the input, the latent representation, and the output at 28 × 28 pixels, the following value was used: kernel, 3 × 3 pixels; padding, 1 × 1 pixels. Abbreviation: CAE, convolutional auto-encoder.

The sizes of the latent representation and the output of CAE were affected by padding and kernel of 2D convolution. To maintain the size of the input, the latent representation, and the output at constant value (28 × 28 pixels), the following value was used: kernel, 3 × 3 pixels; and padding, 1 × 1 pixels. In addition, the following numbers of feature maps were tested: 20, 30, and 40. Typically, CAE is 3-layered neural network. However, a previous study showed that multilayer perceptron with four hidden layers showed better performance than that with two hidden layers [12]. Therefore, CAE with multiple hidden layers was used in the present study (CAE with multiple hidden layers corresponds to conventional convolutional network without a pooling layer). Fig. 3 illustrates the network architecture of CAE with multiple hidden layers. The following numbers of hidden layers were tested: 1, 3, 5, 7, 9, and 11.

The training set was used for training of CAE. Then, the effectiveness of the training was evaluated with the validation set and its MSE. This evaluation was performed for each parameter of CAE, and the best parameters were selected based on the MSE of the validation set.

### 2.6 Application of CAE to image denoising of CT

To denoise ULDCT images, ULDCT image was decomposed into overlapping patches, and each image patch was denoised separately [12]. In the present study, the overlapping image patches were obtained pixel-by-pixel on the ULDCT image, and the image patch was denoised with the best CAE selected in the previous step. After obtaining the denoised patches, the denoised images were obtained by placing these patches and then averaging over the overlapping regions. The CT value was converted using the function [I] before denoising, and inverse transformation of the function [I] was performed after denoising. The testing UDLCT images without image registration were used for assessment of the denoised image.

### 2.7 Other methods of image denoising

The DAE, LNLM, and BM3D methods were tested for comparison with CAE. As shown in the Results section, because the effectiveness of training for DAE was worse than that for CAE, image denoising was not evaluated for DAE. Only MSE of DAE was compared with that of CAE. To train DAE with image patches, the patches of training and validation set were vectorized. After vectorization, the preprocessing using the function [I] was performed before training DAE.

CT images denoised by LNLM and BM3D were compared with those denoised by CAE. For image denoising with LNLM and BM3D, conversion of CT value using the function [I] and inverse transformation of the function [I] were performed as in CAE. For LNLM, the pixel values on denoised image is replaced by the weighted average of pixel values on noisy image located in a neighborhood window. Each weight expresses the similarity in intensity between the central pixel in the window and each neighboring pixel and is given by difference between patch of the central pixel and that of the neighboring pixels [7, 8]. The following parameters were tested for LNLM: patch size, 11 × 11; distance between the patches, 41 × 41 or 81 × 81; h, 0.0001–0.05. In BM3D, image denoising is based on a sparse representation in transform-domain. The enhancement of the sparsity is achieved by grouping similar 2D fragments of the image into 3D group. Then, collaborative filtering is performed, which is developed for dealing with these 3D groups. The collaborative filtering reveals even the finest details shared by grouped fragments and at the same time it preserves the essential unique features of each individual fragment [13]. For the sigma parameter of BM3D, the range 5–40 was tested. In both LNLM and BM3D, the 1st- and 2nd-best parameters were selected from more than 10 combinations of denoising parameters based on visual assessment of the overall quality of the denoised ULDCT image.

### 2.8 Visual assessment of the denoised image

The testing ULDCT images of the upper-, middle-, lower-lung fields were denoised with CAE, LNLM, and BM3D. Five radiologists with 1, 4, 8, 8, and 11 years of experience and three technologists visually assessed the denoised CT images in lung window with a level setting of 1,500/−600 HU. They were unaware of the denoising methods or parameters used. The CT images denoised with CAE, LNLM, and BM3D were visually evaluated side-by-side. The readers independently recorded their subjective impression of the following four items: streak artifacts, noise other than streak artifacts, visualization of pulmonary vessels, and the overall image quality. The denoising methods were ranked by the readers for each image and item. The ranks of the three images (the denoised ULDCT images of upper-, middle-, and lower-lung fields) were summed, and the sum of the ranks of the three images was recorded as the result of the visual assessment. Because each reader selected rank from 1 to 5 for each slice, the result of visual assessment ranged from 3 to 15.

### 2.9 Objective assessment of the denoised image

Peak signal to noise ratio (PSNR) and structural similarity index (SSIM) [18] were used for objective assessment of the denoised image. Because the training set was obtained from CT images of the phantom lungs and subjective evaluation of the denoised image was performed for evaluating the phantom lungs, PSNR and SSIM were calculated using only the pixels of the phantom lungs. For this purpose, pixel values of the SDCT images and the ULDCT images were converted 0 if the pixels did not belong to the phantom lungs. Lung segmentation was performed for SDCT image by using region growing method and threshold = −500 HU [19]. Then, binary closing was applied to the result of lung segmentation to include lung vessel in the result of lung segmentation. The result of lung segmentation was validated visually by one board-certified radiologist (MN). The visually-validated result of lung segmentation was used for calculation of PSNR and SSIM.

### 2.10 Statistical analysis

For each item of visual assessment, the differences in the ranks between various denoising methods were assessed by Mann–Whitney U test. As the readers conducted the visual assessment independently, a statistical test could be performed. *P*-value <0.05 was considered to indicate statistical significance. All analyses were performed by *R*-3.1.1 (available at http://www.r-project.org/).

### 2.11 Extension of our CAE method and additional comparison

Additionally, our proposed method was extended and compared with the method proposed by Chen et al. [16]. Because Chen’s paper was published after our paper was submitted to Heliyon, extension of our proposed method and comparison between our proposed method and Chen’s method was separated from the main experiment.

The following parameters were used for Chen’s method in the current study, because Chen’s paper does not fully show their parameters of convolutional neural network (padding sizes).

• Number of convolution layers was 3. (Number of hidden layers was 2).
• Activation function was Rectified linear units (ReLU).
• Filter numbers (number of feature maps) in the hidden layers were 64 and 32.
• Filter sizes (kernel sizes of convolution) were 9, 3 and 5.
• Padding sizes were 4, 1, and 2.

The network architecture specified by these parameters of Chen’s method can be viewed as a special case of our method shown in Fig. 3. Major differences between Chen’s method and our proposed method shown in Fig. 3 are: (i) the type of activation function (sigmoid vs ReLU), and (ii) sizes of kernel and padding (sizes of kernel and padding were fixed in our proposed method of the main experiment).

From the viewpoint that Chen’s method can be viewed as a special case of our proposed method of the main experiment, we fully optimized the network architecture of Fig. 3. We performed random search [20], and the following parameter space was used for optimizing the network architecture of Fig. 3;

• Numbers of hidden layers were randomly selected from 1, 3, 5, 7, 9, or 11.
• Activation function was ReLU.
• Filter numbers in the hidden layers were randomly selected from 16, 32, 48, or 80.
• Kernel sizes in convolution were randomly selected from 3, 5, 7, 9, or 11.
• Padding sizes were calculated from kernel sizes using the following equation: (padding size) = decimal_truncation((kenel size)/2). For example, if kernel size is 7, then padding size is 3.

In short, we searched the best parameters of numbers of hidden layers, filter numbers, and kernel sizes in this optimization. The random search was performed 100 times, and the best MSE of validation set and its corresponding parameters were selected. In this additional experiment, the same training set and validation set as the main experiment were used in both our proposed method and Chen’s method. ULDCT images denoised with optimal method in the additional experiment were also evaluated as in the main experiment.

## 3 Results

**Fig. 4:**
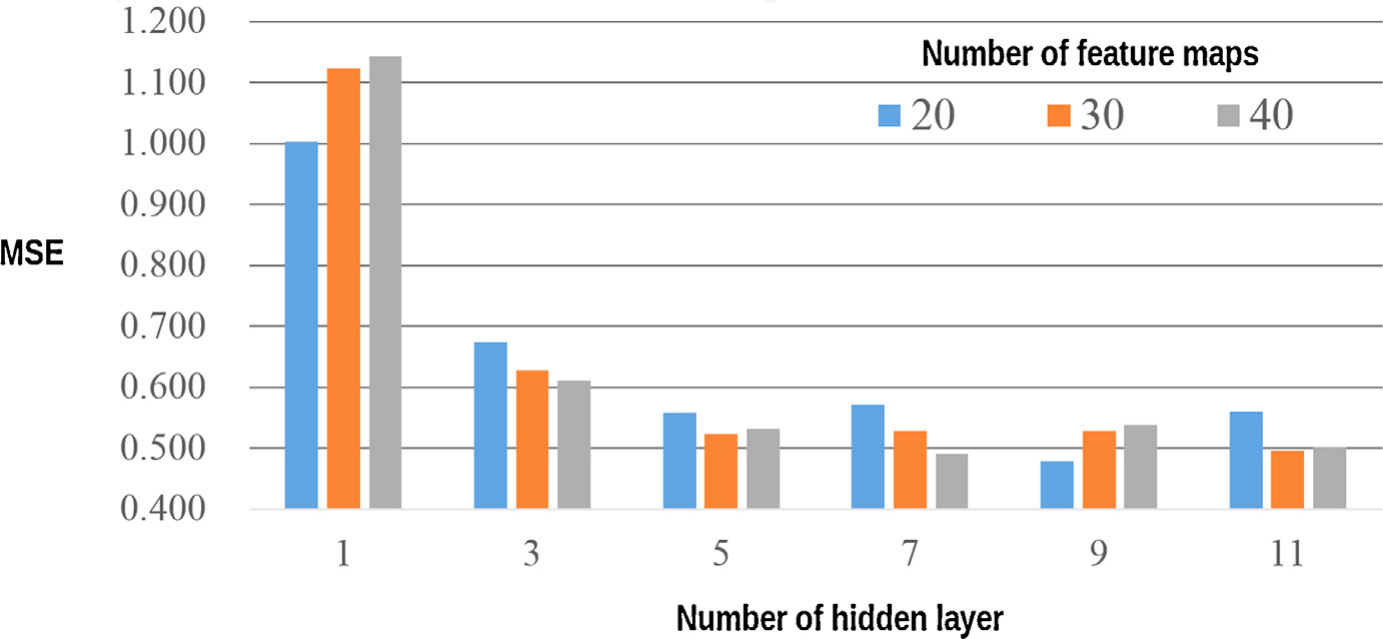
Effect of CAE parameters on MSE obtained from the validation set of image patches. Note: Before denoising the image patches of validation set, MSE between image patches of the SDCT and those of the ULDCT was 2.104. Abbreviation: MSE, mean squared error; CAE, convolutional auto-encoder; SDCT, standard-dose CT; ULDCT, ultra-low-dose CT.

Fig. 4 show the effect of CAE parameters on MSE value obtained from the validation set. As shown, the MSE of the validation set decreased as the number of hidden layers increased. However, when the number of hidden layers was 7–11, the effect of the number of hidden layers was small. In the present study, the best parameters were: number of layers, 9; and number of feature maps, 20. These parameters were used for the image denoising of the ULDCT images.

Table 1 shows the effect of the DAE parameters on MSE obtained from the validation set. While DAE was tested with 1, 3, and 5 hidden layers, the MSE with 5 hidden layers was worse than that with 3 hidden layers. This result suggests that DAE with many hidden layers resulted in vanishing gradients or overfitting. According to Fig. 4 and Table 1, the effectiveness of training in DAE was worse than that in CAE.

**Table 1:** Effect of DAE parameters on MSE obtained from the validation set of image patches.

number of hidden layers	number of hidden units per one layer			
	1000	2000	3000	4000
1	0.827	0.799	0.794	0.788
3	0.663	0.645	0.639	0.640
5	0.999	1.064	1.087	11.345

Note: Before denoising the image patches of the validation set, the MSE between image patches of SDCT and those of ULDCT was 2.104. Abbreviation: MSE, mean squared error; DAE, denoising auto-encoder; SDCT, standard-dose CT; ULDCT, ultra-low-dose CT.

**Fig. 5:**
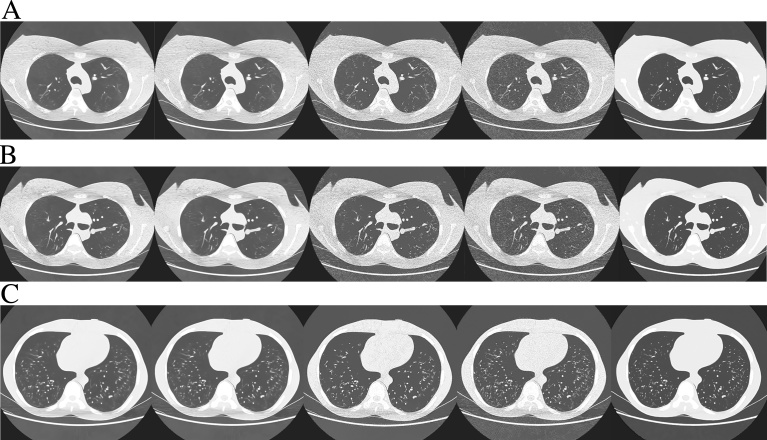
Testing ULDCT images denoised with CAE, LNLM, and BM3D. A, B, and C show the upper-, middle-, and lower-lung fields, respectively. The denoising methods and parameters of these images were: 1st column, BM3D with the parameter of sigma = 25; 2nd column, BM3D with the parameter of sigma = 20; 3rd column, LNLM with the parameter of h = 0.03; 4th column, LNLM with the parameter of h = 0.02; 5th column, CAE. Abbreviation: CAE, convolutional auto-encoder; ULDCT, ultra-low-dose CT; LNLM, large-scale nonlocal means.

**Fig. 6:**
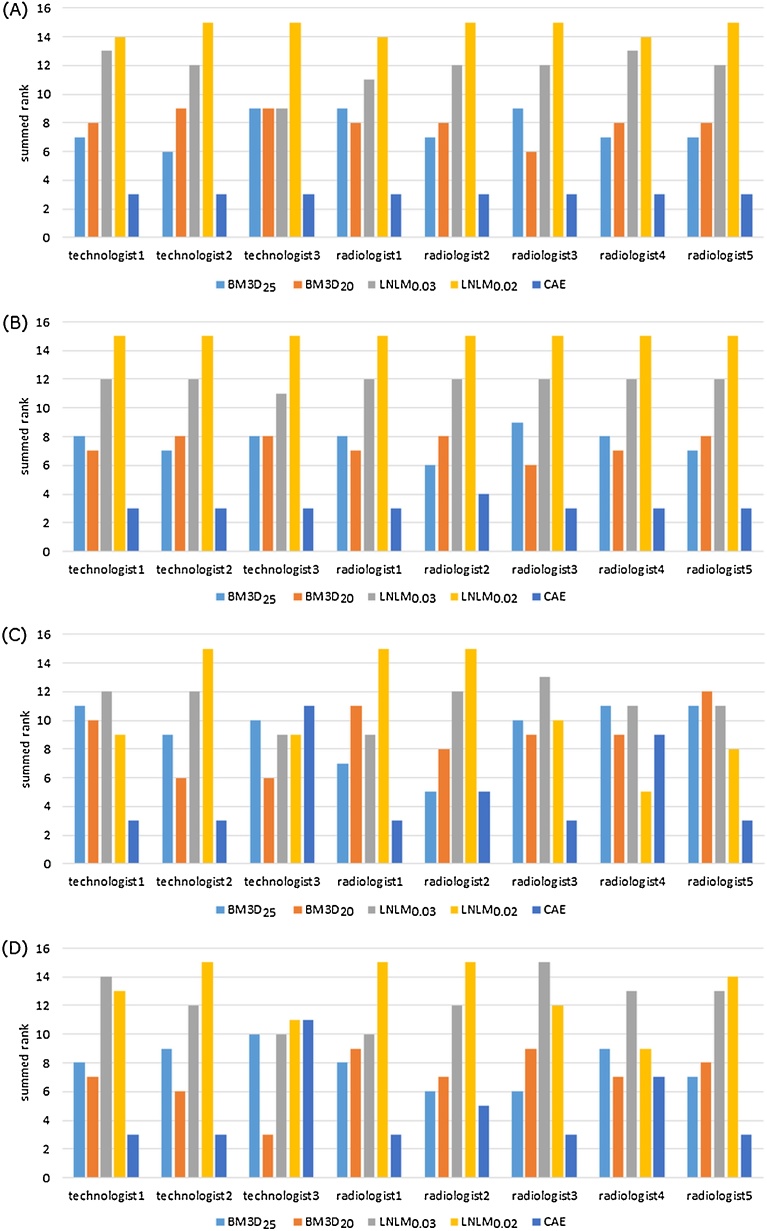
Raw Results of visual assessment of denoised ULDCT images assessed by the 8 readers. (A)–(D), raw results of streak artifacts, noise other than streak artifacts, visualization of pulmonary vessels, and overall image quality, respectively. Note: The sum of the ranks obtained from the three denoised ULDCT images was recorded as the result of the visual assessment. Abbreviation: BM3D_25_, BM3D with the parameter of sigma = 25; BM3D_20_, BM3D with the parameter of sigma = 20; CAE, convolutional auto-encoder; LNLM, large-scale nonlocal mean; LNLM_0.03_, LNLM with the parameter of h = 0.03; LNLM_0.02_, LNLM with the parameter of h = 0.02.

**Fig. 7:**
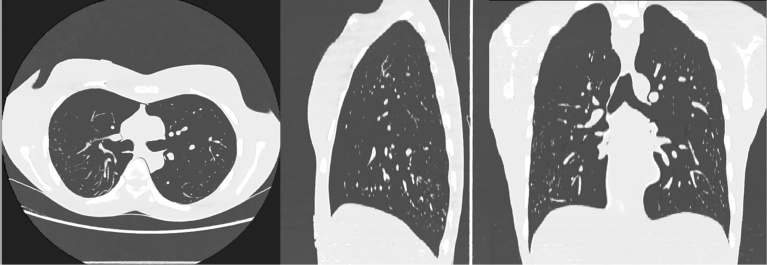
Axial, sagittal, and coronal images of the testing ULDCT which were obtained by denoising with CAE slice-by-slice. Abbreviation: ULDCT, ultra-low-dose CT; CAE, convolutional auto-encoder.

The radiologists and technologists visually evaluated the images of Fig. 5. The following parameters were selected for visual assessment of LNLM and BM3D: for LNLM, patch size = 11 × 11, distance between the patches = 81 × 81, and h = 0.02 or 0.03; for BM3D, sigma = 20 or 25. Table 2 and Table 3 and Fig. 6 show the results of visual assessment of the testing ULDCT images denoised with CAE, LNLM, and BM3D. The raw data of visual assessment are provided in Fig. 6. According to Table 3, except the difference in the overall image quality between BM3D with sigma = 20 and CAE, the p-values between CAE and the other denoising methods were less than 0.05. The results of objective assessment of the ULDCT images are shown in Table 4. Table 4 shows that, as lung CT images, the best PSNR and SSIM were obtained in CAE. Generally, CAE outperformed both LNLM and BM3D. Fig. 7 shows the axial, sagittal, and coronal images of the testing ULDCT, which were denoised slice by slice with CAE.

**Table 2:** Results of visual assessment of the denoised ULDCT images.

Visual assessment		BM3D_25_	BM3D_20_	LNLM_0.03_	LNLM_0.02_	CAE
streak artifact	mean	7.63	8.00	11.75	14.63	3.00
	SD	1.11	0.87	1.20	0.48	0.00
noise other than streak artifact	mean	7.63	7.38	11.88	15.00	3.13
	SD	0.86	0.70	0.33	0.00	0.33
visualization of vessel	mean	9.25	8.88	11.13	10.75	5.00
	SD	2.05	2.03	1.36	3.56	3.00
overall image quality	mean	7.88	7.00	12.38	13.00	4.75
	SD	1.36	1.80	1.65	2.06	2.73

Note: The values of the mean and SD cells were calculated from the results provided by the 8 readers. In the mean cell, the lower value is better. The best is 3, and the worst is 15. Abbreviation: BM3D_25_, BM3D with the parameter of sigma = 25; BM3D_20_, BM3D with the parameter of sigma = 20; CAE, convolutional auto-encoder; LNLM, large-scale nonlocal means; LNLM_0.03_, LNLM with the parameter of h = 0.03; LNLM_0.02_, LNLM with the parameter of h = 0.02; ULDCT, ultra-low-dose CT.

**Table 3:** Results of statistical tests between CAE and other denoising methods.

Visual assessment	BM3D_25_ vs CAE	BM3D_20_ vs CAE	LNLM_0.03_ vs CAE	LNLM_0.02_ vs CAE
streak artifact	0.0003491	0.0003212	0.0003615	0.0003098
noise other than streak artifact	0.0004935	0.0004779	0.0002950	0.0002054
visualization of vessel	0.01785	0.02452	0.003091	0.01356
overall image quality	0.02474	0.07272	0.001622	0.001326

Note: The number is the p-value obtained with Mann-Whitney U test. Except the overall image quality between BM3D_20_ and CAE, the p-values are less than 0.05. Abbreviation: BM3D_25_, BM3D with the parameter of sigma = 25; BM3D_20_, BM3D with the parameter of sigma = 20; CAE, convolutional auto-encoder; LNLM, large-scale nonlocal means; LNLM_0.03_, LNLM with the parameter h = 0.03; LNLM_0.02_, LNLM with the parameter of h = 0.02.

**Table 4:** Results of objective assessment of the ULDCT images.

		PSNR			SSIM	
	upper	middle	lower	upper	middle	lower
Undenoised	58.221	57.593	60.173	0.99791	0.99756	0.99865
BM3D_25_	61.756	60.973	61.485	0.99888	0.99863	0.99892
BM3D_20_	61.753	60.977	61.767	0.99890	0.99865	0.99898
LNLM_0.03_	60.098	59.205	61.091	0.99857	0.99821	0.99886
LNLM_0.02_	58.282	57.646	60.876	0.99794	0.99759	0.99882
CAE	64.634	62.354	62.363	0.99948	0.99900	0.99911

Note: upper, middle, and lower represent upper-, middle-, and lower-lung field, respectively. Abbreviation: BM3D_25_, BM3D with the parameter of sigma = 25; BM3D_20_, BM3D with the parameter of sigma = 20; CAE, convolutional auto-encoder; LNLM, large-scale nonlocal means; LNLM_0.03_, LNLM with the parameter h = 0.03; LNLM_0.02_, LNLM with the parameter of h = 0.02; ULDCT, ultra-low-dose CT; PSNR, peak signal to noise ratio; SSIM, structural similarity index.

For the additional experiment, MSE of the validation set was as follows: optimal method in the main experiment, 0.478; Chen’s method, 0.365; optimal method in the additional experiment, 0.255. The parameters of optimal architecture in the additional experiment were as follows: number of hidden layers, 7; filter numbers in the hidden layers, 16, 64, 48, 64, 80, 48, 32; kernel sizes, 5, 7, 9, 5, 11, 5, 3, 3; padding sizes, 2, 3, 4, 2, 5, 2, 1, 1. According to these results, MSE of Chen’s method is better than that of optimal method in the main experiment. However, by optimizing network architecture shown in Fig. 3, MSE of optimal method in the additional experiment was better than that of Chen’s method. Visual assessment of the denoised CT images was performed for comparison between the 3 methods. According to the results of the one-board radiologist (MN), image quality of these 3 methods was very similar, and it was difficult for the radiologist to rank these 3 methods. Results of objective assessment of the denoised CT images was shown in Table 5. The results show that image quality of ULDCT images denoised with optimal method in the additional experiment was slightly better than that with Chen’s method.

**Table 5:** Results of objective assessment of the ULDCT images for the additional experiment.

		PSNR			SSIM	
	upper	middle	lower	upper	middle	lower
Optimal method in the main experiment	64.634	62.354	62.363	0.99948	0.99900	0.99911
Chen’s method	64.845	62.390	62.648	0.99950	0.99900	0.99920
Optimal method in the additional experiment	64.884	62.464	62.678	0.99951	0.99901	0.99921

Note: upper, middle, and lower represent upper-, middle-, and lower-lung field, respectively. Abbreviation: ULDCT, ultra-low-dose CT; PSNR, peak signal to noise ratio; SSIM, structural similarity index.

## 4 Discussion

The results of the present study show that it was possible to train CAE with pairs of SDCT and ULDCT image patches and to use the trained CAE for patch-based image denoising of ULDCT images. In addition, the results of visual assessment by the radiologists and technologists show that the performance of CAE was better than that of LNLM or BM3D. These results validate the usefulness of image denoising of ULDCT using CAE.

Reduction of radiation dose results in an increase of CT image noise, which is an important factor reducing image quality. Because the high contrast between air and other tissues in lungs helps to maintain diagnostic accuracy, radiologists can reliably evaluate chest LDCT images. In contrast, as severe noise and a significant number of artifacts are present in chest ULDCT images, ULDCT hinders the radiologists’ evaluation. To reduce the noise or artifacts in LDCT or ULDCT images, various types of post-processing techniques have shown potential for reduction of CT radiation dose.

This paper proposes to denoise ULDCT images using CAE. Previously, non-medical images, such as images of human faces, vegetables, and birds have been denoised with neural network [12, 21, 22], and multilayer perceptron or convolutional neural network have achieved good performance for image denoising of non-medical images. For medical image denoising, there are several studies to use conventional neural network [23, 24, 25, 26, 27, 28, 29, 30, 31]. On the other hand, literature survey of Litjens et al. shows that, in deep learning with convolutional neural network or CAE, number of applications of image enhancement like image denoising is limited [32]. Only a few studies show the usefulness of deep learning for image enhancement of CT [16, 33].

Although the previous study shows that multilayer perceptron with four hidden layers performed better than that with two hidden layers [12], Table 1 shows that the neural network with many hidden layers resulted in vanishing gradients or overfitting. CAE was used in the present study to overcome these problems, and our results show that CAE with many hidden layers, corresponding to convolutional neural network without a pooling layer, could be trained successfully. In CAE, the large number of hidden layers resulted in better MSE. These results of the present study validate the usefulness of deep learning with convolutional neural network.

The advantage of CAE is twofold. First, it can be implemented on any CT scanner without using raw CT data. There have been several studies that have showed the usefulness of iterative reconstruction through raw-data-based techniques (e.g., ASIR or SAFIRE) for dose reduction of chest CT [4, 5, 6]. Because iterative reconstruction technique is usable in relatively new CT scanners, the merits of the iterative reconstruction technique cannot be accessed in institutions where older CT scanners are used. This limitation does not exist for post-processing techniques including our CAE method. Second, CAE utilized machine learning to denoise ULDCT images. If training data are plentiful and training of CAE is successfully performed, CAE can automatically optimize an efficient and accurate way to denoise CT images without exact knowledge of the CT scanner model and reconstruction algorithm. In addition, if training data are prepared for each CT parameter (e.g., CT scanner model, radiation-dose level, reconstruction algorithm, and so on), CAE will potentially become useful for automatically optimizing the denoising technique CT images depending on these CT parameters. This scheme would reduce the cost of tuning denoising methods.

The results of visual assessment show that, for noise or artifact reduction, CAE was superior to LNLM or BM3D (p-values < 0.05). Although the difference in the overall image quality between BM3D with sigma = 20 and CAE was not statistically significant (p-value = 0.07272), the image quality of CAE tended to be better than that of BM3D. In addition, the image quality of CAE was significantly better than that of LNLM. These results validate the usefulness of CAE for image denoising of ULDCT images.

There were several limitations in the present study. First, because the present study was experimental, we could not use clinical LDCT or ULDCT images. It is possible that, if patient data is used, our results will not be reproduced. For further study, we would evaluate CAE with the CT images obtained from clinical patients. Second, the raw-data-based techniques were not evaluated in the present study, as the iterative reconstruction technique was not available in the CT scanner of our institution. Therefore, we could not compare CAE with iterative reconstruction technique. If a CT scanner equipped with the iterative reconstruction technique is available, we will compare CAE with the iterative reconstruction technique.

In conclusion, the current study proposed image denoising with CAE, which was trained with the patches of SDCT and ULDCT images. After training of CAE, ULDCT images could be reliably denoised. According to visual assessment by radiologists and technologists, the performance of CAE was superior to that of LNLM and BM3D.

## Declarations

### Author contribution statement

Mizuho Nishio: Conceived and designed the experiments; Performed the experiments; Analyzed and interpreted the data; Contributed reagents, materials, analysis tools or data; Wrote the paper.

Chihiro Nagashima, Saori Hirabayashi, Akinori Ohnishi, Kaori Sasaki, Tomoyuki Sagawa, Masayuki Hamada, Tatsuo Yamashita: Performed the experiments.

### Funding statement

This work was supported by JSPS KAKENHI (Grant Number JP16K19883) and Hyogo Science and Technology Association.

### Competing interest statement

The authors declare no conflict of interest.

### Additional information

No additional information is available for this paper.
